# A Rapid, Accurate, and Efficient Method to Map Heavy Metal-Contaminated Soils of Abandoned Mine Sites Using Converted Portable XRF Data and GIS

**DOI:** 10.3390/ijerph13121191

**Published:** 2016-12-01

**Authors:** Jangwon Suh, Hyeongyu Lee, Yosoon Choi

**Affiliations:** 1 John & Willie Leone Department of Energy and Mineral Engineering, The Pennsylvania State University, State College, PA 16802, USA; jangwonsuh@hanmail.net; 2 Korea Environment Institute, Sejong 30147, Korea; lhg6197@gmail.com; 3 Department of Energy Resources Engineering, Pukyong National University, Busan 48513, Korea

**Keywords:** soil contamination mapping, portable X-ray fluorescence (PXRF), geographic information systems (GIS), ordinary Kriging, heavy metal contamination, abandoned mine

## Abstract

The use of portable X-ray fluorescence (PXRF) and inductively coupled plasma atomic emission spectrometry (ICP-AES) increases the rapidity and accuracy of soil contamination mapping, respectively. In practice, it is often necessary to repeat the soil contamination assessment and mapping procedure several times during soil management within a limited budget. In this study, we have developed a rapid, inexpensive, and accurate soil contamination mapping method using a PXRF data and geostatistical spatial interpolation. To obtain a large quantity of high quality data for interpolation, in situ PXRF data analyzed at 40 points were transformed to converted PXRF data using the correlation between PXRF and ICP-AES data. The method was applied to an abandoned mine site in Korea to generate a soil contamination map for copper and was validated for investigation speed and prediction accuracy. As a result, regions that required soil remediation were identified. Our method significantly shortened the time required for mapping compared to the conventional mapping method and provided copper concentration estimates with high accuracy similar to those measured by ICP-AES. Therefore, our method is an effective way of mapping soil contamination if we consistently construct a database based on the correlation between PXRF and ICP-AES data.

## 1. Introduction

Mine tailings generally contain several types of potentially toxic elements (PTEs) and can widely be dispersed by percolating rainwater or mechanical transport in runoff. Enriched PTEs in soil not only cause soil contamination but influence human health via the food chain [1,2,3,4,5]. To minimize the risk that arises from the high level of PTEs in soil, an examination of the extent and the level of contamination based on environmental assessment and monitoring is an important first step towards appropriate remediation [6,7]. The Korean Ministry of Environment (MOE) Standard suggests using an inductively coupled plasma atomic emission spectrometry (ICP-AES) analysis method to investigate the type and content of heavy metals in soil due to its high accuracy [8]. However, ICP-AES has a few disadvantages, such as non-portability, high operating costs, and lengthy period required for elemental analysis due to complex preprocessing (e.g., an acid digestion of the sample is needed when analyzing soil samples) [9,10,11,12]. These time and cost constraints can reduce the amount of accessible data that affects the quality of the soil contamination mapping.

To compensate for the disadvantages of ICP-AES, an in situ analysis method using a portable X-ray fluorescence (PXRF) instrument has been widely used. The U.S. Environmental Protection Agency’s (EPA’s) Method 6200 specifies the use of PXRF to examine the types and levels of PTEs in soil [13,14]. Although a method using PXRF has the advantage of analyzing the sample in a short period of time on site, thereby providing abundant data, it has the disadvantages of high uncertainty and relatively low accuracy [15,16,17,18]. For these reasons, a soil contamination mapping method using the analysis data from either PXRF or ICP-AES still has limitations in exploring spatial distributions of PTEs.

It is certainly difficult to deny the fact that the use of PXRF has facilitated rapid soil contamination mapping, while ICP-AES has enabled accurate soil contamination mapping. However, soil contamination mapping using either the PXRF or ICP-AES method has disadvantages in terms of accuracy or rapidity. Little efforts have made to incorporate advantageous aspects of both methods into the investigation of soil contamination. In practice, despite their high cost, repetitive multiple generations of soil contamination mapping over extensive regions are necessary during the comprehensive soil management period, from the identification of PTEs to the remediation and follow-up monitoring of contaminated soil. Thus, the development of a rapid, inexpensive, and accurate soil contamination mapping method is necessary to address this problem.

Many recent studies worldwide have sought to create soil contamination maps to identify the spatial distribution of PTEs in soil [10,17,19,20,21,22,23,24,25]. Most of these studies used ICP-AES to investigate the types and levels of PTEs in soil samples collected on site. However, because of the aforementioned disadvantages of ICP-AES, these studies either required more than seven days to be completed or were limited by the difficulty in securing sufficient elemental concentration data to create a soil contamination map over the entire region.

To overcome these limitations, some studies generated soil contamination maps using PXRF analysis data. Tolner et al. [10] studied the characteristics related to the results of an in situ PXRF analysis of soil samples such as the transport pathway of point sources of pollutants, the influence of water content in the soil sample on the change in the detected level of heavy metals, and estimation errors according to the measurement time. Higueras et al. [24] examined the time required to investigate soil contamination of large-scale mine areas using PXRF and proved that the PXRF analysis method can be effectively utilized to determine the reclamation priority. Carr et al. [17] reported a case study demonstrating the effectiveness of using PXRF and geographic information systems (GIS) to map the contaminated soils. Lee et al. [11] compared the prediction performances of different approaches, such as the application of ordinary Kriging and co-Kriging methods to PXRF and ICP-AES data to generate soil contamination maps for copper and lead. However, these previous studies had certain limitations: (1) Relatively less accurate PXRF data without any correction were utilized as input data for geostatistical spatial interpolation; (2) Additional sampling based on the judgment of an on-site investigator was not performed after initial sampling; and (3) The total time required for the entire soil contamination mapping process from the selection of the sampling point to the visualization of the heavy metal content was not analyzed, although a few studies assessed only the duration of the elemental analysis of soil samples by PXRF [16,24].

To address and overcome these limitations, this study aims to develop a rapid and accurate soil contamination mapping method using in situ PXRF analysis data corrected by ICP-AES data and GIS. The proposed method includes an efficient selection of sampling locations, rapid investigation and effective compilation of collected data, securement of the accuracy of PXRF data, visualization of soil contamination data, and evaluation of PTE transport. The proposed method was applied to an abandoned metal mine in Korea and validated for investigation rapidity and prediction accuracy. The distinct difference from the previous study [11] is that this work focuses on developing an efficient investigation and mapping procedure for heavy metal contamination, not on comparing the performances of geochemical analysis equipment or geostatistical spatial interpolations.

## 2. Study Area

The study area included a segment of the abandoned metal mine located at Kijang-gun, Busan, South Korea (129°13′25.57′′ E, 35°18′31.36′′ N). Abandoned in 1990, this mine was one of the largest copper mines in South Korea between 1938 and 1945 and produced copper, gold, silver, and tungsten. With no proper environmental remediation after the mine’s closure, high concentrations of PTEs were identified in the soil because of the large amount of mine waste rock and leakage of underground mine water containing toxic heavy metals.

In 1999, the Korean government initiated a mine reclamation project to prevent this problem. However, soil contamination had already been broadly dispersed because of the continuous leakage of PTEs. In particular, copper concentrations in the study area greatly exceeded the Korean Contamination Warning Standard of 150 mg/kg [8] to levels that can pose a risk to human health [26]. Therefore, copper was selected as the target element among various PTEs detected in the soil of the study area.

## 3. Methods

**Figure 1:**
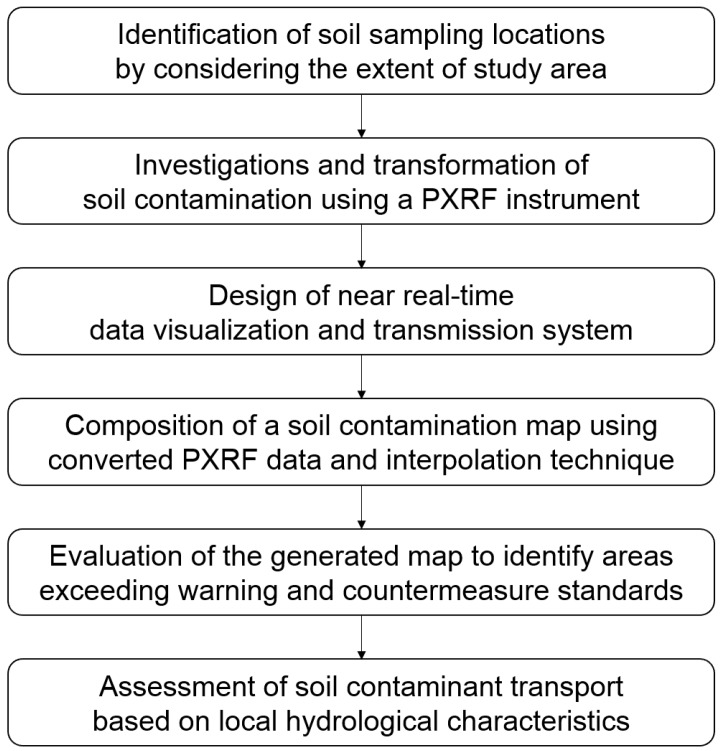
Procedure for the rapid, accurate, and efficient soil contamination mapping method developed in this study.

In this study, a rapid and accurate method of generating a soil contamination map that compensates and overcomes the aforementioned limitations of the previous studies was developed using in situ PXRF elemental analysis data and spatial interpolation techniques. The method consists of five steps shown in Figure 1.

### 3.1. Selection of Soil Sampling Locations by Considering the Extent of the Region of Interest

It is very important to select appropriate sampling locations because the selection can directly influence the results of the soil contamination mapping to be used to identify spatial distributions of PTEs. In this study, selection of sampling points was conducted twice (before and after the initial survey) by considering the extent of the study region and the possibility of soil sampling and accessibility. The location of the pollution sources was not considered in the selection of sampling points because it was uncertain that they would be able to be distinguished in soils.

**Figure 2:**
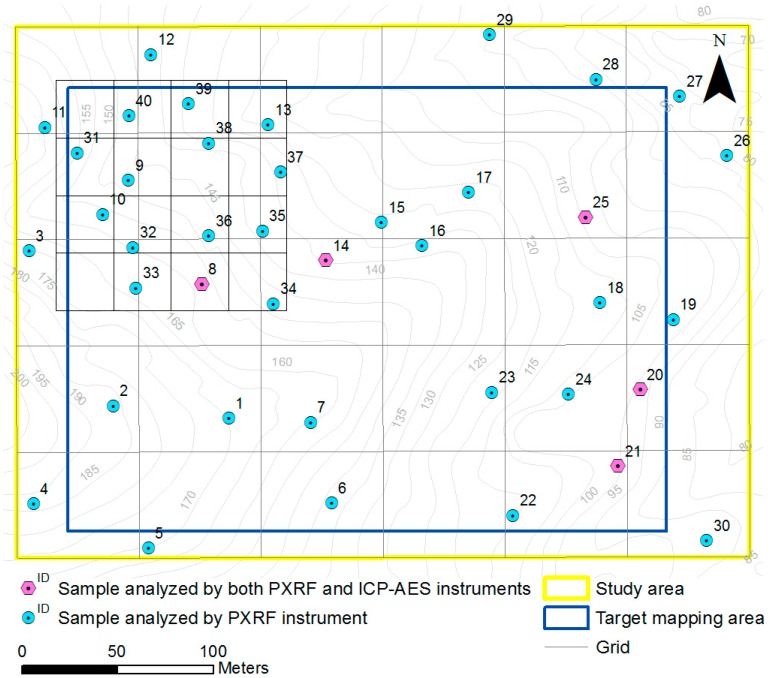
Sampling points for soil contamination survey using portable X-ray fluorescence (PXRF) and inductively coupled plasma atomic emission spectrometry (ICP-AES).

In the first investigation, a regular grid with a width of 60 m was created over the entire study area to roughly analyze the general distribution of copper concentrations (Figure 2) and avoid uneven distribution of sampling locations by ensuring that each square of the grid contained at least one sampling point. The distribution of sampling points is relevant to the results of the PTE concentrations predicted by geostatistical spatial interpolation [27]. These sampling intervals are denser than that of previous studies that employed ICP-AES according to the Korean Standard Test (KST) method for the examination of the spatial distribution of PTEs in soil [28]. Initially, 30 points were selected as soil sampling locations within the grid to examine the general spatial distribution of the study area. After examining the general pattern of the copper content by in situ elemental analysis using PXRF, the site investigator selected ten additional soil sampling locations in the vicinity of anomaly regions (northwest region of the study area) within a smaller grid with a width of 10 m. A large dataset will result in a more accurate delineation of the anomaly regions where engineering remediation is necessary. To validate the accuracy of the concentration estimates, soil samples were collected at five randomly chosen points for both PXRF and ICP-AES analyses (Figure 2). Due to the inherent characteristics of the interpolation method used to generate an accurate soil contamination map, the extent of the target mapping area was set to be smaller than that of the original study area to reduce prediction errors or uncertainties about the boundary of the mapping area. Although most previous studies have not applied this strategy, it is crucial to conduct a second sampling campaign using the results of the initial survey since understanding the extent and the level of contamination in highly contaminated areas is a priority for mine reclamation.

### 3.2. Analysis and Transformation of Heavy Metal Concentrations in Soil Using Portable X-ray Fluorescence (PXRF)

Prior to surveying the study area, as a preliminary investigation, the copper content of 11 soil samples obtained from a previous study conducted in other Korean Cu- and Pb-abandoned mines [11] (not collected at this site) was analyzed using PXRF and ICP-AES to determine the correlation between PXRF and ICP-AES analysis data and examine the accuracy and tendency of the concentration data analyzed by PXRF. The copper content of the 11 samples analyzed by PXRF ranged from 29 to 3068 mg/kg [11]. Since the range of copper concentrations in the previous study was wider than that of the current study, the correlation obtained in the previous study can be applied to the current study areas. Numerous cases of trend equations for the 11 soil samples were considered to minimize errors and maximize the coefficient of determination factor (R^2^).

**Figure 3:**
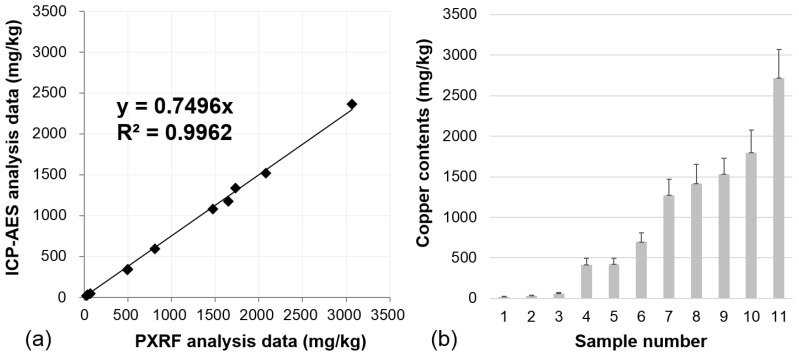
Plot showing the (**a**) Correlation between ICP-AES and PXRF values for Cu; (**b**) Standard deviation of the copper concentration in each point.

The resulting trend equation (ICP-AES analysis value = 0.7496 × PXRF analysis value) indicated that the PXRF analysis data, with relatively low accuracy, tended to be higher than that of ICP-AES. This tendency was also identified in the previous study [11]. Since the ICP-AES method includes an extraction with 0.1 M HCl that only extracts available copper as opposed to performing a full soil digestion, some copper is likely to be bound to acid-resistant minerals and not likely to be measured. In contrast, the XRF method measures all copper in the sample. This equation enabled the transformation of PXRF data values with low accuracy into their corresponding ICP-AES data values with high accuracy. In addition, the coefficient of determination factor (R^2^) between the PXRF and ICP-AES data was computed as 0.9979 for copper (Figure 3a), indicating a very strong correlation between the two datasets. Standard deviation of the copper concentrations between the trend line and each point was low compared with the copper concentration values obtained by PXRF analysis (Figure 3b). As such, 11 soil samples were sufficient to derive the trend equation. This coefficient value for copper was larger than that calculated by another PXRF instrument, Metorex X-MET 920-P model, used by the U.S. EPA [29].

The soil samples were preprocessed and analyzed as follows: For the PXRF analysis method, after removing the impurities such as weeds and organic matter, topsoil was sampled down to 10 cm in depth using a hand auger. The metal content detected would be approximately 1%–3%, 23%–30%, and 30%–39% lower when the water content was 10%, 15%, and 20%, respectively compared to the metal content of completely dried soil [10]. Hence, the water content in each soil sample was measured using a portable soil moisture meter (PMS-714, Lutron, Taiwan). The elemental analysis by PXRF was then conducted when the soil sample water content was less than 10%. Soil samples with less than 10% water content were sieved to particles less than 2 mm in the field to have a homogeneous fraction for precise analysis using PXRF. The prepared soil samples were analyzed as a loose powder for 60 s by PXRF (Innov-X DELTA handheld XRF analyzer developed by Olympus (Tokyo, Japan)), using a sample cup fitted with a 6 μm-thick polyester film. This PXRF instrument is equipped with an Au anode as the excitation source and a silicon drift detector, and operates at 40 kV and 0.1 mA [11]. The quantification was conducted by the software embedded in the PXRF instrument. The Compton normalization method was used as a calibration method and it offers speed, ease of use, and relatively high accuracy for wide concentration ranges.

For the ICP-AES analysis, after being air-dried at 25 °C in the laboratory for five days, the soil samples were disaggregated and sieved to particles less than 2 mm in size, and then ground to a fine powder (<2 µm). According to the KST method for the chemical analysis of soils [8,30], soil samples were digested with 0.1 N of HCl solution, with 10 g of soil in 50 mL of the solution. The samples were then heated at 60 °C for 30 min and subsequently at 140 °C for 90 min. After cooling and filtration, the solutions were diluted to 100 mL with distilled water. The samples were analyzed for copper content using ICP-AES (VISTA-PRO developed by Varian, Palo Alto, CA, USA). The quantification of copper by ICP-AES was performed using the measured intensity of atomic emission at the wavelength of 324.75 nm and a calibration curve according to the KST method. A blank test was conducted for the calibration.

The copper content of the soil at 40 sampling points was analyzed using PXRF. The analyzed data were then transformed using the equation of the best fit line displayed in Figure 3a. The results were compared using an independent validation dataset derived by ICP-AES analysis at five points where PXRF analysis was previously conducted. The validated and converted PXRF copper concentration data were then used as input for spatial interpolation.

### 3.3. Design of Data Visualization and Transmission Systems in near Real-Time

**Figure 4:**
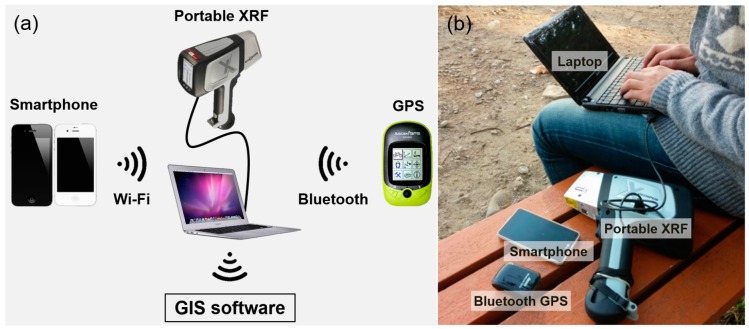
Near real-time data transmission system. (**a**) Conceptual diagram; (**b**) Photo of instruments.

This study developed a system for visualizing and transmitting in situ elemental analysis data using PXRF. Figure 4a shows a descriptive diagram for the near real-time data transmission system and Figure 4b shows a photo of the equipment used for data acquisition and transmission. This system included four pieces of equipment: a PXRF, a portable Bluetooth GPS (global positioning system) receiver, a smartphone, and a laptop. The Bluetooth GPS receiver was used to record the geographical coordinates of the sites where PXRF analysis was conducted in real-time. The simultaneous use of PXRF and the Bluetooth GPS facilitated the acquisition of georeferenced elemental composition data in soils. In addition, since georeferenced contamination data are exported in a comma-separated value (CSV) format, they can be presented in any commonly used form of spreadsheet. A smartphone was used to establish a wireless communication network via its mobile tethering function for data transmission in near real-time. This enabled the site investigator to transmit the collected data to be shared in near real-time via a personal cloud storage service such as Google Drive or Dropbox.

**Figure 5:**
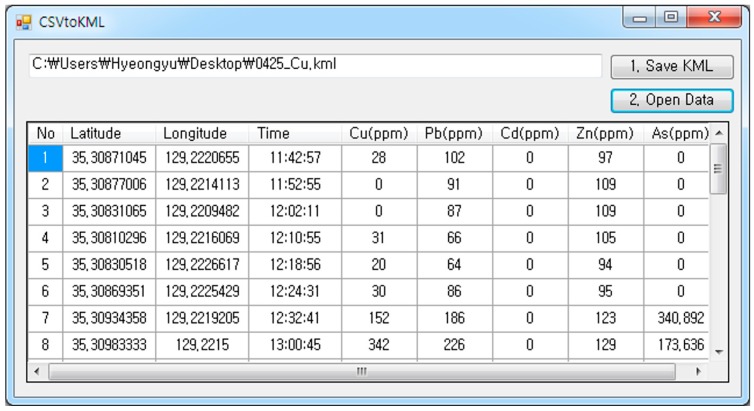
Interface of the comma-separated value (CSV) to Keyhole Markup Language (KML) program developed in this study.

With the aim of the effective and rapid visualization of the distribution of the heavy metal concentration data at the site or in the office, a software was developed using the Visual Basic.NET programming language (Microsoft, Redmond, WA, USA). The software was designed to provide a spreadsheet-type interface for data input and convert CSV-formatted output data into the Keyhole Markup Language (KML) format required to visualize the data on Google Earth (Figure 5). When the created KML file is executed, one or more pinpoints with attribute tables including date and time of data acquisition, latitude, longitude, and concentrations of elements are displayed at each sampling location on Google Earth. The default color of the pinpoint is green; however, the pinpoint is shown automatically either in yellow or red when the copper concentration exceeds either the Korean Contamination Warning Standards (150 mg/kg) or the Korean Contamination Countermeasure Standards (450 mg/kg) announced officially by the Korean MOE, respectively. The Korean Contamination Warning Standards indicate the levels at which PTEs may begin to affect the health of humans, animals, and plants. The Korean Contamination Countermeasure Standards show the levels of PTEs that require immediate action or measures to reclaim the contaminated soil.

### 3.4. Generation of a Soil Contamination Map

The best way to identify the spatial variability of heavy metal content in soil is to analyze as many samples as possible over the entire study area using a high accuracy ICP-AES instrument, although it is very difficult in practice to do so due to time and cost constraints. Therefore, in general, a grid-based soil contamination map is created by applying a geostatistical spatial interpolation method to limited amount of data. To obtain better estimates from the interpolation, a large amount of data is required for the model. The converted PXRF analysis data obtained using the correlation between PXRF and ICP-AES data was used in this study to produce accurate elemental concentration data. The converted PXRF data were then used as input for soil contamination mapping to estimate copper concentrations for the entire target area. This approach made it possible to overcome the problem of data insufficiency and achieve rapidity and accuracy of soil contamination mapping by incorporating the advantages of both PXRF and ICP-AES.

Since both PXRF and ICP-AES can provide data on PTEs only at certain points and not for the entire region, it is generally difficult to explore the spatial distribution of PTEs for an abandoned mine site. The production of soil contamination maps requires the estimation of concentrations at unsampled locations using limited data and interpolation techniques. As such, we considered Kriging, a common interpolation method used in geostatistics, to predict copper concentrations in soil over the entire grid in this study. Kriging is a geostatistical technique which estimates values at unknown locations by considering both the distance and the degree of variation among known data points.

(1) $$z^{*}=\overset{N}{\underset{i=1}{\sum }}\lambda_{i}z_{i}$$

In Equation (1), z* is the Kriging-estimated value at the unsampled point, z_i_ is the known value of spatially distributed data at the sampling point, λ_i_ is the weight of each data, and N is the total number of data used for the Kriging estimation. According to the method that determines the weight, λ_i_, Kriging can be classified into the following categories: simple Kriging, ordinary Kriging, universal Kriging, and co-Kriging.

Ordinary Kriging is the most common geostatistical estimator [31]. It is mainly used when data satisfy a weak secondary invariance and do not show a particular trend. It considers local variations in the mean by limiting the domain of stationarity of the mean to the local neighborhood. In estimating an unknown value using ordinary Kriging, the error variance is minimized while the Kriging estimation equation is not biased. Bias is defined as the difference between the factor average of the population and the estimation equation average for predicting that population factor: when there is no difference, it is considered unbiased. The condition for the equation not to become biased is described as follows:

(2) $$b_{z^{*}}=E\left(z\right)-E\left(z^{*}\right)=E\left(z\right)-E\left(\overset{N}{\underset{i=1}{\sum }}\lambda_{i}z_{i}\right)=0$$

where b_z*_ shows a bias of z*, and since all data used in Kriging have an identical average value, the sum of the weights must be 1. This is shown in the following equation in order for the Kriging estimation equation to always be unbiased:

(3) $$1-\;\overset{N}{\underset{i=1}{\sum }}\lambda_{i}z_{i}=0$$

Ordinary Kriging was selected as the spatial interpolation technique in this study for two reasons: First, a previous comparative study [11] revealed that ordinary Kriging was the most appropriate method among the several Kriging methods to estimate the spatial pattern of PTEs in soil. Second, the soil contamination distribution of copper in this study area satisfies a weak secondary invariance and does not show a particular trend.

### 3.5. Identification of Areas with Hazardous Soil Contamination

As many PTEs are known to pose a risk to human health, their presence in high concentrations in soil is clearly a matter of concern. To assess and manage the risk of soil contamination with elevated levels of PTEs, the Korean MOE established the Korean Contamination Warning Standards and the Korean Contamination Countermeasure Standards according to the type and risk level of each heavy metal [8]. Based on these standards, contaminated soil areas were classified into three groups: areas with levels below warning standards, areas with levels exceeding warning standards, and areas with levels exceeding countermeasure standards. These two standards were applied to the soil contamination map for copper generated in this study to identify highly contaminated areas where preventative measures against the spread of soil contamination were necessary. Subsequently, the area of the contaminated region was calculated since it is needed for planning of reclamation budget and establishment of post-closure management.

### 3.6. Evaluation of Potentially Toxic Trace Element (PTE) Transport Based on Local Hydrological Characteristics

The spatial variations of heavy metal enrichment in soil may be related to natural dispersion processes [17]. In particular, transport or spatial distribution of PTEs in an abandoned mine area are considerably affected by local topography and surface runoff [32,33,34,35,36,37]. Accordingly, the contribution of hydrological characteristics based on the local topographical features on the spatial pattern of copper concentration was examined using a digital elevation model (DEM) in this study. For the generation of a DEM, a triangulated irregular network (TIN) model was created using topographic contour lines obtained from topographic maps with a scale of 1:5000. TIN was then converted to a grid-based DEM with a spatial resolution of 3 m showing the distributions of terrain relief. Spurious depressions on the DEM were identified by ground inspection and were removed by using the Fill tool in ArcGIS 10.1 software (ESRI, Redlands, CA, USA). Subsequently, the flow directions (determined by the direction of steepest descent, or maximum drop, from each grid cell) of surface runoff in the watershed zones were analyzed using the DEM and the hydrology tools in ArcGIS. By comparing the locations of sampling points and the local flow direction pattern, the effect of flow direction of surface runoff on the transport and dispersion of copper was evaluated.

## 4. Results

**Figure 6:**
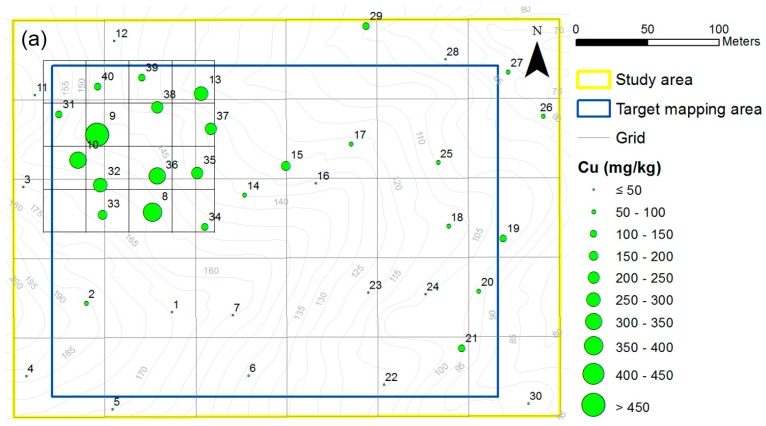

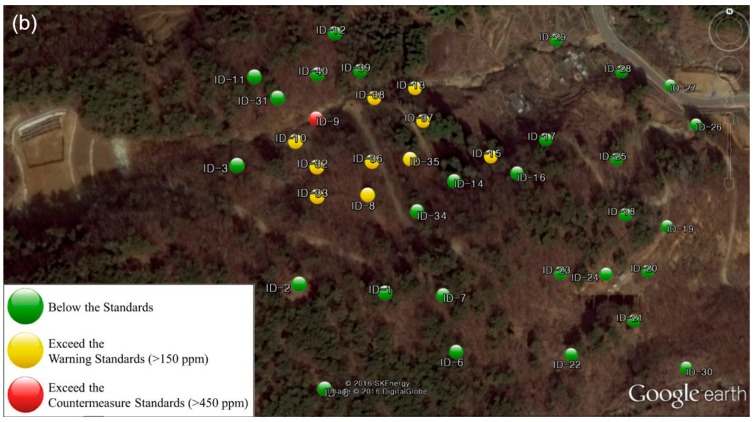
Results of PXRF elemental analysis. (**a**) Distribution of Cu concentration; (**b**) Google Earth screenshot of distribution of Cu content according to the environmental risk countermeasure criteria.

Figure 3a shows the copper concentration (mg/kg) results in the soil at each point obtained using PXRF. Using the trend equation between PXRF and ICP-AES data seen in Figure 3a, 40 raw data points with low accuracy (copper content measured by PXRF) were transformed into converted PXRF data with higher accuracy (close to the ICP-AES analysis value). The results showed large variations in the concentrations over the sampling locations with copper concentrations ranging from 13.5 to 717.4 mg/kg. Among the 40 soil samples, 11 samples exceeded 150 mg/kg (the Korean Contamination Warning Standards for copper) and one sample exceeded 450 mg/kg (the Korean Contamination Countermeasure Standards for copper). The spatial distribution of the converted PXRF data (Figure 6a,b) shows that high levels of copper in soil were most abundant in the northwestern part of the study area. It took approximately 5 h to determine the copper content in the soil at 40 sampling points.

**Figure 7:**
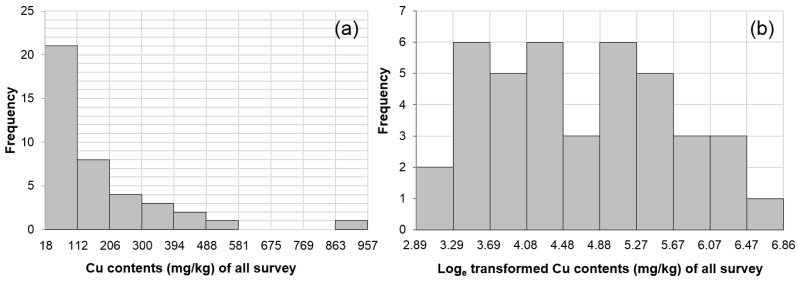

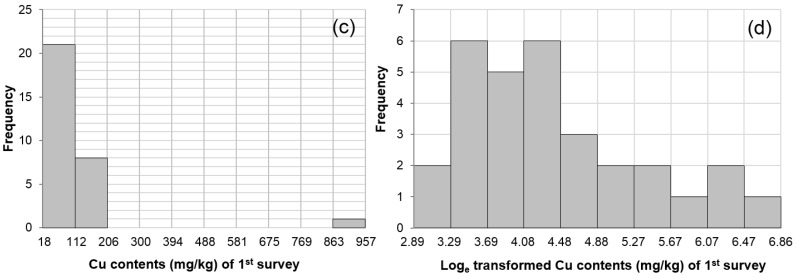
Histograms of copper content analyzed with the PXRF instrument. (**a**) All raw data of copper by PXRF; (**b**) Natural logarithms of the all raw data; (**c**) 30 raw data samples of copper by PXRF without second survey; (**d**) Natural logarithms of the 30 raw data samples without second survey.

Summary statistics of the converted PXRF analysis results reveal the following values for copper concentrations: maximum = 717.4 mg/kg, minimum = 13.5 mg/kg, mean = 128.22 mg/kg, standard deviation = 137.66 mg/kg, and skewness = 2.28. The converted PXRF data were positively skewed, as seen in the histogram in Figure 7a, suggesting that it did not follow a normal distribution. For ordinary Kriging interpolation, however, input data should follow a normal distribution to reduce the effect of the extreme values in estimating the target value. Therefore, all the converted PXRF data for copper were transformed to their natural logarithms and they mostly followed a normal distribution (Figure 7b). However, the data did not follow a normal distribution without the 10 data points additionally analyzed in the second survey (Figure 7c,d). Thus, it was inappropriate to apply ordinary Kriging interpolation to the sampled data obtained only from the first survey conducted to examine general spatial distribution of Cu content in soil. This is why additional sampling based on the judgment of an investigator on site after initial sampling is necessary.

In situ data measured by PXRF and Bluetooth GPS were transmitted four times to other investigators on site and to a researcher in the office via a laptop and a smartphone to share the data with them in near real-time. Consequently, the researchers in the office effectively identified operation statuses and outcomes in near real-time via developed data visualization and transmission systems. The visualization of the shared KML data on Google Earth as well as the data spreadsheet file format made it possible to intuitively understand the status of the measurements and the spatial variability of the copper content in the soil (Figure 6b). Furthermore, the data visualization system of preliminary soil contamination helped the site investigator in near real-time select additional sampling locations (near ID-9 of Figure 6b) based on their own judgment or on discussion with other researchers who were not at the site.

**Figure 8:**
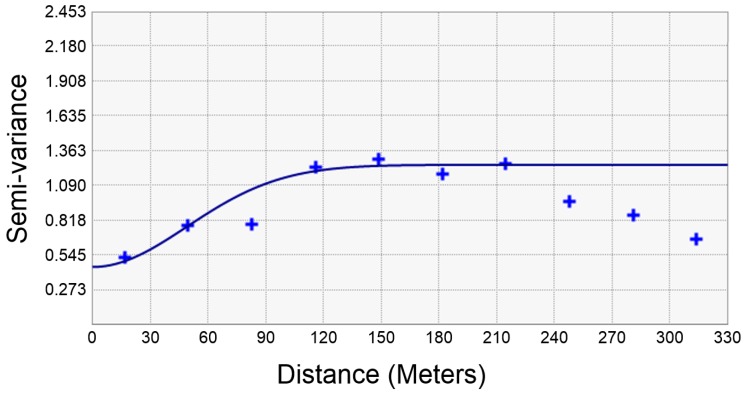
Experimental variogram modeling of Cu content in the study area.

To derive the spatial correlation of the transmitted data, variogram modeling was performed (Figure 8). Several lag distance conditions were considered to examine the theoretical variogram, which fits the overall pattern of an experimental variogram. A Gaussian model was selected at a fitting function for theoretical variogram. An isotropy model was selected since there was no distinct anisotropy in the converted PXRF analysis data. The ordinary Kriging method was applied to 40-point data because the data satisfied a weak secondary invariance and did not show a particular trend. Variogram modeling parameters of nugget, sill, and range were set as 0.45, 0.8, and 120, respectively, for the generation of the soil contamination map.

**Figure 9:**
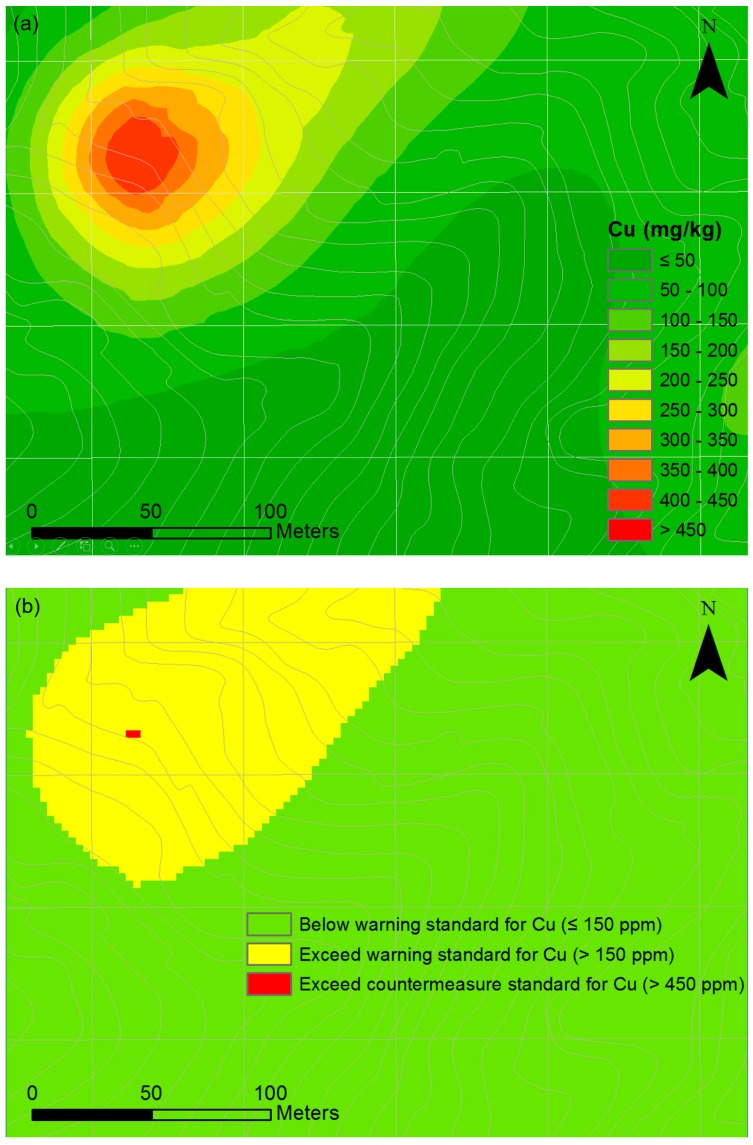
Soil contamination map. (**a**) Spatial distribution map of Cu; (**b**) Areas with high levels of soil contamination exceeding the Korea Soil Contamination Warning Standards or Countermeasure Standards.

A soil contamination map of estimated elemental concentrations over the entire target area was generated for copper by applying the ordinary Kriging method (Figure 9a). There were large variations in the copper concentrations over the target area, with the highest concentrations in northwestern part of the target area. The areas with levels exceeding the warning standards and countermeasure standards were calculated to be 14,049 m^2^ and 18 m^2^, respectively (Figure 9b). As a first step, these areas required additional sampling and detailed assessment of the samples to delineate the exact extent of the highly contaminated areas. Subsequently, appropriate remediation measures against the spread of soil contaminants should be carried out based on the generated soil contamination map to prevent risks to human health.

**Figure 10:**
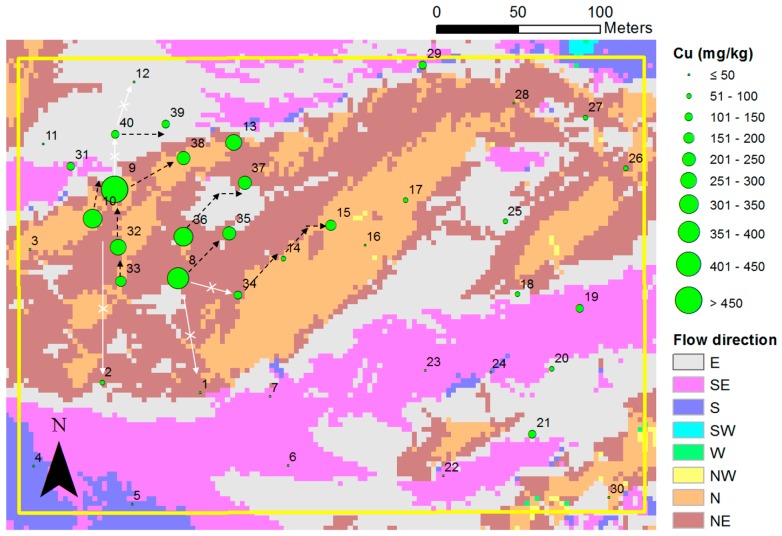
Hydrological analysis for the evaluation of the effect of flow direction of surface runoff on the copper dispersion.

Figure 10 shows the flow direction of surface runoff of each grid cell and copper concentrations at the sampling points in the target mapping area. We estimated that the contaminants would spread downslope based on the result of the generated map. This was similar to the flow direction of surface runoff (dotted arrow in black). Based on the local topographic relief, the spreading pattern of soil contaminants was influenced by the flow direction of rainwater, although we could not determine the distinct pollution sources in the study area. If high-level contaminants located in the northwestern part of Figure 10 were transported to the south (white arrow), the soil sample No. 1 and 2 might have shown higher copper concentrations. This finding can assist in selecting additional sampling points for further investigation or validation.

## 5. Discussion

The time duration and prediction accuracy for the soil contamination mapping method were validated to assess the method’s performance in terms of efficiency and reliability.

### 5.1. Analysis of Time Duration

The time required for each process of the soil contamination mapping is summarized in Table 1. The comprehensive site investigation work, including movement, soil sample analysis, data transmission, mapping, and hazardous area filtering, was carried out on 25 April 2015, from 9 a.m. to 5:30 p.m. It took 230 min to analyze copper concentrations at 40 sampling points and approximately 80 min to transmit the analyzed data including the breaks. Roughly 27 min was spent for the final mapping of soil contamination. Lastly, it took 10 min to evaluate the transport of PTEs using the DEM-based hydrological analysis approach. Soil contamination mapping conducted solely using data obtained from ICP-AES, which is the conventional method commonly used in the geochemistry field, may take at least seven days [8]. From this standpoint, the developed method using PXRF can help substantially reduce the time required to compose a soil contamination map to 5% of the time required for the conventional method. Accordingly, the developed mapping approach can take the place of the conventional mapping method in terms of time efficiency by constructing a database on the correlation between PXRF and ICP-AES analysis data for a certain element.

### 5.2. Validation of Converted PXRF Data

**Figure 11:**
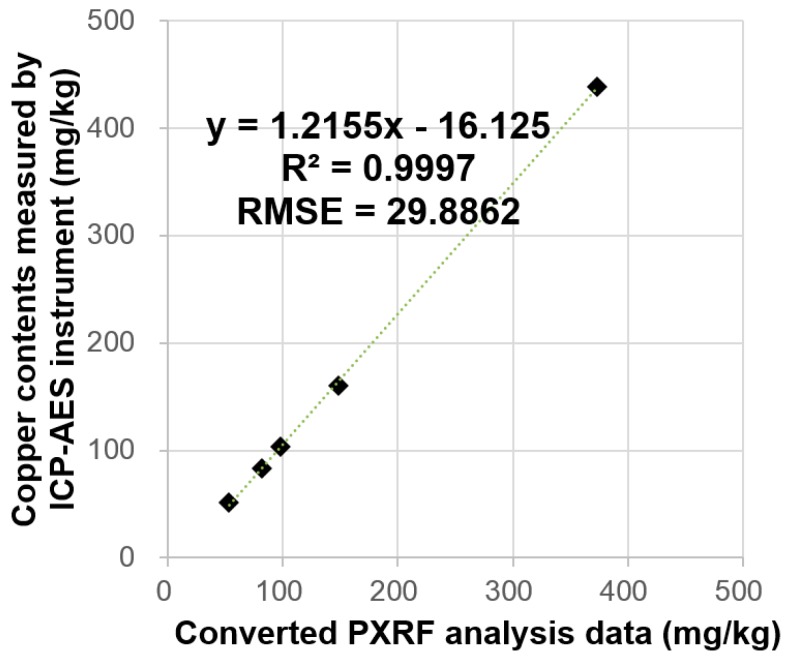
Plot showing correlation between transformed PXRF analysis values and ICP-AES analysis values for the five validation samples.

To validate the applicability of the correlational equation between PXRF and ICP-AES data from Figure 3 to convert PXRF data obtained in this study, ICP-AES analysis for copper was conducted at five randomly selected sampling points where PXRF analysis had previously been implemented (Table 2). Subsequently, the prediction performance of the converted PXRF data was compared with that of the ICP-AES data at these five identical sampling points. The ratio of the number of validation data to the number of sampling data is 12.5% in this study. This ratio is similar to that (12%) of a previous study [11] that compared the PXRF (training set) and ICP-AES data (validation set). The result of the comparison of the two datasets is shown in Figure 11. The graphs show that the gradient (slope) of the first-order trend line and y-intercept were determined to be 1.2155 and −16.125, respectively. The root mean square error (RMSE) was calculated to be 29.8862. With five validation data points, the slope was not significantly different from 1; however, the y-intercept was not close to 0. This is due to the sample ID 8 in Table 2 which had a value highly distant from the other sample values. When this sample (ID: 8) was excluded, the slope was 1.1461, the y-intercept was −9.7980, and RMSE was 6.5526. Compared with the case of five validation data points, the slope was closer to 1, the y-intercept was closer to 0, and the RMSE was smaller. These results indicate that there are minor differences between the two datasets. In addition, the converted PXRF data were as accurate as the ICP-AES data in this study. Thus, the mapping method developed in this study can be effectively used to estimate the level and extent of soil contamination in terms of prediction accuracy. However, the difference in copper concentrations was relatively higher when concentrations were elevated indicating that the correlation between these two methods for highly contaminated samples may be not as high as for mildly contaminated samples. Hence, this result should be taken into account for future analysis or for the comparison of the converted PXRF data with ICP results.

## 6. Conclusions

This study proposed a rapid and accurate soil contamination mapping method using converted portable X-ray fluorescence (PXRF) data and geostatistical spatial interpolation, and analyzed the prediction accuracy and time required for mapping copper concentrations in soil. A soil contamination map for copper was developed by applying the mapping method to 40 points in the vicinity of an abandoned metal mine. Regions with areas of 14,049 m^2^ and 18 m^2^ were found to exceed Korean Contamination Warning Standards and Countermeasure Standards for copper in soil, respectively, both of which point to the soils that require remediation. A comparison with the conventional soil contamination mapping method using inductively coupled plasma atomic emission spectrometry (ICP-AES) showed that, in terms of practicality, the developed method can considerably reduce the time (by 95%, only approximately 8 h needed for collecting and analyzing 40 samples) required for implementing the entire mapping process (i.e., selection of sampling locations, in situ data measurement and analysis, data transmission, mapping, and interpretation). Our method also provides high-accuracy estimates of copper concentrations in soil (R^2^ = 0.9997) with relatively low-cost converted PXRF data and only five ICP-AES data. Therefore, the method developed in this study can be used as a very efficient soil contamination mapping procedure in terms of investigation speed, operating costs, and estimation reliability.

Additional sampling points near highly contaminated sites should be selected using the preliminary soil contamination map when creating grid-based soil contamination maps by geostatistical interpolation with limited data for two reasons: (1) to provide a dataset following a normal distribution for the geostatistical interpolation (with only one-time general sampling, the site may not follow a normal distribution as presented in Figure 7) and (2) to precisely delineate the extent of highly hazardous zones exceeding warning or countermeasure standards where mine reclamation measures are necessary to minimize the risk to human health.

Although the target element was confined to copper in this study, the in situ soil contamination mapping method can also be applied to the areas contaminated with other potentially toxic trace elements (PTEs), i.e., zinc, lead, and arsenic. However, since the detection limit for PTEs of the PXRF instruments is relatively high compared with that of ICP-AES instruments, the estimation accuracy for the content of trace elements and the quality of the resulting contamination map are expected to be low. Therefore, it is beneficial to utilize both PXRF and ICP-AES data to generate a contamination map and to explore the spatial variability of soil contamination according to the types and levels of contaminants.

If we have sufficient in situ PXRF data and a database of correlations between PXRF and ICP-AES analyses for a certain element, validation with ICP-AES analysis can be omitted, thereby simplifying the mapping procedure and providing a better option in terms of rapidity and accuracy in generating high-quality soil contamination maps. To construct a reliable correlation database or a trend equation for the elemental concentrations measured by the two methods, it would be appropriate to use soil Standard Reference Materials (SRM) as well as in situ soil samples. Moreover, there are numerous types of PXRF instrument models worldwide with different levels of performance. Accordingly, the construction of the database on the correlation between PXRF and ICP-AES analysis data for PTEs based on the PXRF instrument model is necessary for improving the method of in situ soil contamination mapping.

## Figures and Tables

**Table 1:** Time duration for each task.

Start	End	Duration	Task
09:00 a.m.	10:00 a.m.	60 min	Identification of sampling locations
10:00 a.m.	11:12 a.m.	72 min	Movement and preparation
11:12 a.m.	12:02 p.m.	50 min	1st investigation (7 samples)
12:02 p.m.	12:30 p.m.	28 min	Data transmission, visualization and break
12:30 p.m.	01:45 p.m.	75 min	1st investigation (12 samples)
01:45 p.m.	02:03 p.m.	18 min	Data transmission, visualization and break
02:03 p.m.	03:05 p.m.	62 min	1st investigation (11 samples)
03:05 p.m.	03:19 p.m.	14 min	Data transmission, visualization and break
03:19 p.m.	04:00 p.m.	41 min	Preliminary soil contamination mapping
04:00 p.m.	04:43 p.m.	43 min	2nd investigation (10 samples) for the additional sampling locations
04:43 p.m.	05:30 p.m.	47 min	Data transmission and final soil contamination mapping
05:30 p.m.	05:40 p.m.	10 min	Interpretation of PTE transport using DEM-based hydrological analysis
Cumulative duration		8 h 40 min	

**Table 2:** Results of elemental analysis for Cu content using an ICP-AES instrument for the validation of transformed PXRF data.

Sample ID	Cu (mg/kg)			
	Raw PXRF	Transformed PXRF	ICP-AES	Standard Deviation
8	498	373.30	438.83	46.3155
14	109	81.71	82.92	0.8485
20	131	98.20	103.23	3.5355
21	198	148.42	160.35	8.4853
25	71	53.22	51.59	1.1314

## Abbreviations

The following abbreviations are used in this manuscript:

PXRF	Portable X-ray fluorescence
ICP–AES	Inductively coupled plasma atomic emission spectrometry
PTEs	Potentially toxic trace elements
MOE	Ministry of Environment
GIS	Geographic information systems
GPS	Global positioning system
CSV	Comma-separated value
KML	Keyhole Markup Language
DEM	Digital elevation model
TIN	Triangulated irregular network
